# β-Amyloid Precursor Protein Does Not Possess Ferroxidase Activity but Does Stabilize the Cell Surface Ferrous Iron Exporter Ferroportin

**DOI:** 10.1371/journal.pone.0114174

**Published:** 2014-12-02

**Authors:** Bruce X. Wong, Andrew Tsatsanis, Linh Q. Lim, Paul A. Adlard, Ashley I. Bush, James A. Duce

**Affiliations:** 1 Oxidation Biology Unit, The Florey Institute of Neuroscience and Mental Health, The University of Melbourne, Parkville, Victoria, Australia; 2 School of Molecular and Cellular Biology, Faculty of Biological Sciences, University of Leeds, Leeds, West Yorkshire, United Kingdom; 3 Department of Pharmacology and Therapeutics, The University of Melbourne, Parkville, Victoria, Australia; 4 Department of Pathology, The University of Melbourne, Parkville, Victoria, Australia; Torrey Pines Institute for Molecular Studies, United States of America

## Abstract

Ceruloplasmin is a ferroxidase that interacts with ferroportin to export cellular iron, but is not expressed in neurons. We recently reported that the amyloid precursor protein (APP) is the analogous iron-exporting chaperone for neurons and other cells. The ferroxidase activity of APP has since been called into question. Using a triplex Fe^2+^ oxidation assay, we analyzed the activity of a soluble form of APP (sAPPα) within a buffer of physiological pH and anionic charge, and determined that iron oxidation originated from phosphate. Using various techniques such as flow-cytometry to measure surface presented proteins, we confirmed that endogenous APP is essential for ferroportin persistence on the neuronal surface. Therefore, despite lacking ferroxidase activity, APP still supports iron export from neurons.

## Introduction

Since its discovery, much of the research of the type 1 transmembrane protein β-amyloid precursor protein (APP) has focused on its proteolytic components, particularly the β-amyloid (Aβ) peptide that accumulates in Alzheimer’s disease. However, full length APP is yet to be attributed a conclusive function. It has been described to have roles in transcriptional signaling, synapse formation, ion transport, neuroprotection and neuroplasticity [1]. Recently we have added to this growing functional list by reporting that both the full-length membrane bound and the cleaved soluble extracellular form of sAPPα, but not other family members amyloid precursor-like protein (APLP) 1 and 2, facilitate the efflux of iron from APP-expressing cells such as neurons [2].

As an integral cofactor in many metabolic processes, iron must be closely regulated for the wellbeing of any cell, particularly where oxygen consumption is high such as in the neuron. The ability for iron to undergo redox-cycling is harnessed by some enzymes for catalysis [3], however under aerobic conditions iron may also catalyze the production of reactive oxygen species (ROS) through the Haber-Weiss [4] and Fenton [5] reactions. Unregulated hydroxyl radical (OH•) and ROS production is damaging to the cell [6] and have been associated with aging and disease, particularly in neurodegenerative diseases such as Alzheimer’s disease, Parkinson’s disease and aceruloplasminemia, where iron accumulates in affected tissue [7].

As both iron deficiency or excess may compromise cell viability, homeostasis is tightly controlled with cell entry, storage and exit [8]. Import of iron was previously considered to be solely through either divalent metal transporter 1 (DMT1) [9] or by transferrin import through interaction with the Transferrin Receptor (TfR) [10]. However, other import mechanisms have now been described including ZIP14 [11], indicating that uptake of iron into the cell may not be as simple as previously thought. Currently, there is only one known iron export pore protein, ferroportin (FPN), which is believed to traffic Fe^2+^ from the cytoplasm to the plasma membrane surface. While a variety of mechanisms are thought to facilitate the release of iron from the exofacial surface of FPN, multicopper ferroxidases such as ceruloplasmin (CP), hephaestin (Heph) and the bacterial ferroxidase Fet3, were previously considered the only facilitators of intracellular iron efflux. This was mediated through their ability to secure Fe^2+^ from stabilized FPN on the cell surface and promote Fe^2+^ oxidation for Fe^3+^ loading into iron-transporting proteins such as transferrin (TF) [12]–[14]. We concluded that APP might also fulfill an analogous function for iron release [2].

We found that the major proportion of APP in human and mouse post-mortem brain tissue samples is complexed to FPN, and that APP knockout mice markedly accumulate iron in several organs, including the brain [2]. Several reports have since corroborated the impact of APP expression on cellular iron levels [15]–[18]. This is likely to be the mechanism by which sAPPα is neuroprotective against glutamate excitotoxicity, and a point mutation within the REXXE motif within APP, a site common within other iron homeostatic proteins, negates this neuroprotection [2]. A peptide fragment of APP containing this motif was recently reported to interact with FPN and stabilize it on the surface of human brain microvascular endothelial cells [19].

We also reported that APP could catalyze the oxidation of Fe^2+^ through a mechanism we thought analogous to ferritin, a ferroxidase that does not have a multicopper active site [2]. Concerns have since arisen about the validity of this chemistry, and other groups were unable to show similar activity when using regions within APP containing the ferritin-homologous REXXE motif that we originally suggested was required for this activity [19]–[21]. Here we re-examine the mechanism for APP-promoted iron export, and whether APP enzymatically catalyzes the oxidation of Fe^2+^. Based on established assays used extensively to monitor iron oxidation, we developed a more reliable assay system [22] that enable studies in an environment that takes into account the physiological levels of phosphate (0.80–1.45 mM [23], [24]), and transferrin (25.5–45.0 µM [25]). We confirm that APP stabilizes surface FPN, and that it is indeed unable to catalyze ferroxidation, which therefore appears to be irrelevant to its ability to facilitate cellular iron export.

## Materials and Methods

### Reagents

Reagents were all analytical grade and were purchased from Sigma (Australia), unless otherwise stated. Purified human ceruloplasmin was purchased from Vital Products (USA). Recombinant full-length human APP with a C-terminal fused Fc region of human IgG was purchased from Sino Biological Inc. (China). FPN was detected by antibodies, gifted by Prof. Tracy Rouault, raised to epitopes in either an extracellular (MAP23; α/α165–181) or intracellular (MAP24; α/α240–258) region of the protein.

### Recombinant protein preparation

Recombinant human sAPP695α was expressed in the methylotrophic yeast Pichia pastoris strain GS115 and purified from culture media by FPLC (BioRad) as previously described [26]. Chromatographic purification was by anion exchange using Q Sepharose (1.6×25 cm column, GE Healthcare) followed by hydrophobic exchange with phenyl Sepharose (1.6×25 cm column, GE Healthcare). APP eluted in phenyl Sepharose buffer B (50 mM Na_2_HPO_4_, pH 7) was then concentrated using Amicon Ultra-15 Centrifugal Filter Units (30 kDa, Merck Millipore, Australia) and stored at –80°C as 20 µM stocks. Elimination of anionic buffer in original sAPPα, APP, CP and BSA stocks was carried out by buffer-exchange with 50 mM HEPES, 150 mM NaCl, pH 7.2 (HBS) using a Superdex 200 10/GL filtration column (GE Healthcare).

### Direct and indirect enzymatic measurement of Fe^2+^ oxidation

Three previously established procedures that either directly or indirectly spectrophotometrically measure catalyzed oxidation of Fe^2+^ to Fe^3+^ have recently been combined and adapted to a plate assay [22]. Assaying the consumption of Fe^2+^ (“Ferrous Loss Assay”) was adapted from Erel [27] and measured the colorimetric change at absorbance 590 nm when the chromagen 3-(2-pyridyl)-5,6-difurylsulfonic acid-1,2,4-triazine sodium salt (Ferene S) is bound to ferrous ions compared to ferric ions. Fe^3+^ production was directly measured by the absorbance (310 nm) of Fe^3+^ (“Ferric Gain Assay”) as previously described by Minotti and Ikeda-Saito [28]. Finally, the physiological loading of apo-TF (apo-TF) with Fe^3+^, which induces an absorbance increase at 460 nm, was assayed using a procedure adapted from that originally reported by Johnson *et al.* [29] and used in the original investigation on apparent ferroxidation promoted by APP [2]. All of these reactions were performed at 24 °C so that the reactions would proceed more slowly to enable measurement with standard spectrophotometers [22].

To a 200 µL reaction, the protein of interest (500 or 250 nM), or media, was added to HBS (50 mM HEPES, 150 mM NaCl, pH = 7.2) ± apo-TF (50 µM). Ferrous ammonium sulfate ((NH_4_)_2_Fe(SO_4_)_2_·6H_2_O; 100 µM) was added to start the reaction. Absorbance readings (310 nM and 460 nM) were kinetically monitored with the Flexstation 3 (Molecular Devices) or PowerWave HT (BioTek) microplate spectrophotometer over 20 min at 30 s intervals with continual agitation and kept at 24°C. Ferene S (500 µM) was added at the end of 20 min and absorbance (590 nm) read. Reactions were blanked against HBS only and all dilutions are shown as final concentrations.

### Preparation of mouse primary neuronal cultures

All animal procedures were approved by the Howard Florey Institute animal ethics committee (#12-078; Melbourne, Australia), and were carried out in accordance with the Australian code of practice for the care and use of animals for scientific purposes as described by the National Health and Medical Research Council of Australia. Pregnant mice were deeply anesthetized with isofluorane and then sacrificed by cervical dislocation, without suffering. Primary neurons cultures from the cortices of wild-type embryos were prepared from embryonic day 14 or 15 mice, as previously described [30]. Cortices were removed, dissected free of meninges, and dissociated in 0.025% trypsin. Cortical cells were plated onto poly-L-lysine (50 µg/ml)-coated 6-well plates (Nunc) at a density of 600,000 cells/cm^2^ in DMEM supplemented with 10% fetal bovine serum (FBS; PAA Laboratories), 5% horse serum and 10 µg/ml gentamycin sulfate. The neurons were allowed to adhere for 2–3 h before the plating medium was replaced with Neurobasal-supplemented medium (serum free and with B27 minus antioxidants, 500 µM glutaMAX and 10 µg/ml gentamycin sulfate). On the day of the iron experiments the medium was replaced with fresh Neurobasal-supplemented medium and for all further experimentation the medium was serum-free.

### Surface Biotinylation assay

Parental HEK293T cells, which constitutively express APP [31] but not CP [2], [14], were maintained in Opti-MEM media with 10% FBS. 48 hours after plating, cells were treated with serum-free Opti-MEM media (Life Technologies). Where required, HEK293T or wild-type mouse primary neurons cells were incubated with ferrous ammonium sulfate (FAS; 50 µM) for 3 h before washing with their respective serum-free media and then replaced with serum-free media ±1 µM sAPP695α or CP for 30 min, prior to surface biotinylation using the Pierce cell surface protein isolation kit (Thermo Scientific).

The procedure for cell surface biotinylation and isolation was carried out as per the manufacturer’s instructions and at 4°C to minimize protein internalization. In brief, cells were labeled with Sulfo-NHS-SS-Biotin in PBS for 30 min. Unreacted biotin was quenched by the Quenching solution provided, before cells were harvested by scraping and lysed using Lysis buffer+Phosphatase Inhibitor I and II (1∶1000). Cell lysates were clarified by centrifugation at 10,000 × g for 2 min and a sample of the supernatant was taken to measure protein levels in the ‘total homogenate’ fraction. To precipitate biotinylated surface proteins from each cell lysate, 200 µg of total protein was incubated with NeutrAvidin Agarose for 1 hour. Non-bound ‘intracellular’ proteins were separated by centrifugation (1,000 × g for 1 min) before agarose was washed three times, and bound proteins eluted with XT sample buffer (Bio-Rad) containing 50 mM DTT, followed by analysis by western blotting.

From each experimental condition total homogenate lysate starting material (10 µg protein), as well as samples of both non-biotinylated (‘intracellular’) and biotinylated (surface) eluates from the 200 µg of lysate separated by NeutrAvidin Agarose were separated on 4–20% PAGE (Bis-Tris, Invitrogen) and transferred to PVDF membrane using a Transblot (Bio-Rad). Primary antibodies used were rabbit anti-FPN (1∶1000, MAP23 recognizing residues 165–181 or MAP24 recognizing residues 240–258 [32], kind gift from Tracey Rouault), mouse anti-APP (1∶1000, WO2 recognizing residues 672–699 [33] or 22C11 recognizing residues 66–81 [34], in house), and rabbit anti-Cp (1∶5000, Dako; #Q0121). The load control was either mouse anti-β-actin (1∶5000, Sigma) or mouse anti-Na/K ATPase (1∶5000, Sigma). Proteins were visualized with ECL (Amersham) and a LAS-3000 Imaging suite, and analyzed using Multi Gauge (Fuji). Densitometry using Image J (v1.48k, NIH) was performed in triplicate on 3 separate experiments. All quantitation was standardized against β-actin and Na/K ATPase levels and secondary antibody alone was used to eliminate the risk of non-specific binding to proteins on the PVDF membrane.

### Fluorescence-activating cell sorting analysis

Parental N2a neuroblastoma cells, were maintained in DMEM media (Lonza) containing 10% FBS until required. For siRNA transfections, cells were washed once with OptiMEM and incubated for 30 min at 37°C in fresh OptiMEM. During this incubation, APP (SMARTpool: ON-TARGETplus Human APP siRNA; Fisher Scientific Ltd) and scrambled (ONTARGETplus Nontargeting Pool; Fisher Scientific Ltd) siRNA were prepared at a final concentration of 50 nM in siRNA buffer (Thermo Scientific) and OptiMEM to a volume of 1 mL. In turn, this was added to 1 mL of OptiMEM containing 20 µL of Dharmafect 1 (Fisher Scientific Ltd), prepared separately. After 15 min incubation, 8 mL of DMEM containing 10% FBS was added to the 2 mL mixture. OptiMEM media was removed from cells and an appropriate volume of siRNA was added. Cells were incubated with the siRNA mixture for 36 hours at 37°C, 5% CO_2_ prior to fluorescence-activated cell sorting (FACS) analysis.

Prior to the experiment, cells were pre-incubated with ferric ammonium citrate (50 µM) in OptiMEM for 6 hours in order to generate sufficient surface FPN expression to be detected on the subsequent FACS, to test whether this would be suppressed by RNAi of endogenous APP or increased by the subsequent 30 min addition of 1 µM sAPP695α in OptiMEM without iron. RNAi under baseline conditions (no additional iron) was not feasible because FPN immunoreactivity was just above the limit of detection, and an experimental treatment that might suppress FPN surface expression would be difficult to exhibit clearly. FACS preparation involved washing and collection of cells in PBS (without Ca and Mg) (Lonza) at room temperature. After cells were pelleted by centrifugation at 2000 rpm for 5 min the remaining procedure was performed at 4°C. Cells, re-suspended in ice-cold FACS buffer (PBS, 2.5 mM EDTA pH 8.0), were incubated with a primary antibody against N-terminal APP (1∶50; Abcam; ab15272) or FPN (1∶50; BioScience Life Sciences) for 30 min. FPN and APP antibodies used were raised to epitopes on extracellular domains and intensity of fluorescence was compared to cells stained with secondary only to minimize the detection of non-specific binding. Cells were washed with FACS buffer before lightly fixing with 1% paraformaldehyde (Alfa Aesar) for 4 min. Cells were then re-suspended in FACS buffer containing AlexaFluor 488 goat anti-rabbit IgG secondary antibody (1∶200; Life Technologies) for 30 min in the dark. Cells were further washed with FACS buffer before incubating with DAPI (Cell Signaling Technology) to differentiate dead cells. Cells were sorted by forward and side scatter on a BD-LSR-Fortessa (BD Biosciences) with a 488 nM blue laser according to fluorescence at 530±30 nM. Minimums of 10,000 cells were recorded in each experiment, having gated the cell population to ensure that only live cells were monitored. Experiments were carried out in duplicate on 3 separate occasions and data were analyzed using BD FACS DiVa 6 and FlowJo 7.6.4 software.

### Confocal Microscopy

A coverslip was placed at the bottom of each well of a 24-well plate, on which wild-type mouse primary neurons were incubated at 50% confluency for 14 days in neuroblastoma media. Where required, cells were incubated with FAS (50 µM) for 3 h before the medium was replaced with fresh neurobasal-supplemented medium ±1 µM sAPP695α or CP for 30 min, prior to immunofluorescence staining. After each experimental condition, cells were washed with cold PBS and then fixed with 4% paraformaldehyde for 4 min at room temperature. Non-permeabilized cells were rinsed again in cold PBS before incubation with blocking buffer (5% (v/v) BSA) for 1 h at 4°C. Cells were then incubated with the appropriate primary antibodies diluted in blocking buffer for a further 2 h at 4°C. Primary antibodies used were rabbit anti-FPN (1∶100, MAP23) and mouse anti-APP (1∶50, 22C11). Cells were then incubated with the fluorescently conjugated secondary antibodies for 1 h in blocking buffer after further washes in PBS. Alexa Fluor 488 Goat anti-Rabbit IgG and Alexa Fluor 488 Goat anti-Mouse IgG (Millipore) were used in combination at 1∶500. Cells were washed a final time before counterstaining with DAPI (1∶1000) and mounted onto slides using fluoromount G mounting medium. A Leica SP8 confocal microscope was used to collect z-stacks of neurons, deconvoluted using the Huygens deconvolution software (Scientific Volume Imaging), and each stack compiled using Image J software.

### Statistical Analysis

Statistical analysis was performed with Microsoft Excel 2011 and GraphPad Prism v5.0 software. Primarily, analysis was carried out with a 2-tailed t-test with the level of significance set at *P* = 0.05.

## Results

### Evaluating the in vitro ferroxidase activity of APP

We revisited the *in vitro* experiments testing sAPPα ferroxidase activity previously presented as part of our primary publication [2]. In a physiologically relevant environment the enzymatic activity of sAPPα was again compared to the established ferroxidase, CP. A triplex assay we have recently developed from existing assays [22] was utilized to kinetically appraise Fe^2+^ oxidation and the loading of Fe^3+^ into TF simultaneously at a physiological pH and with minimal auto-oxidation interference.

For reevaluating the enzymatic activity of APP, human sAPPα was expressed and purified from yeast as per Henry *et al’*s original purification procedure [26]. This recombinant protein was comparable to our original report [2] and to others (e.g. [35], [36]). In addition, a further source of human recombinant full-length APP expressed in bacteria was commercially obtained to corroborate findings using our in-house purified sAPPα.

**Figure 1:**
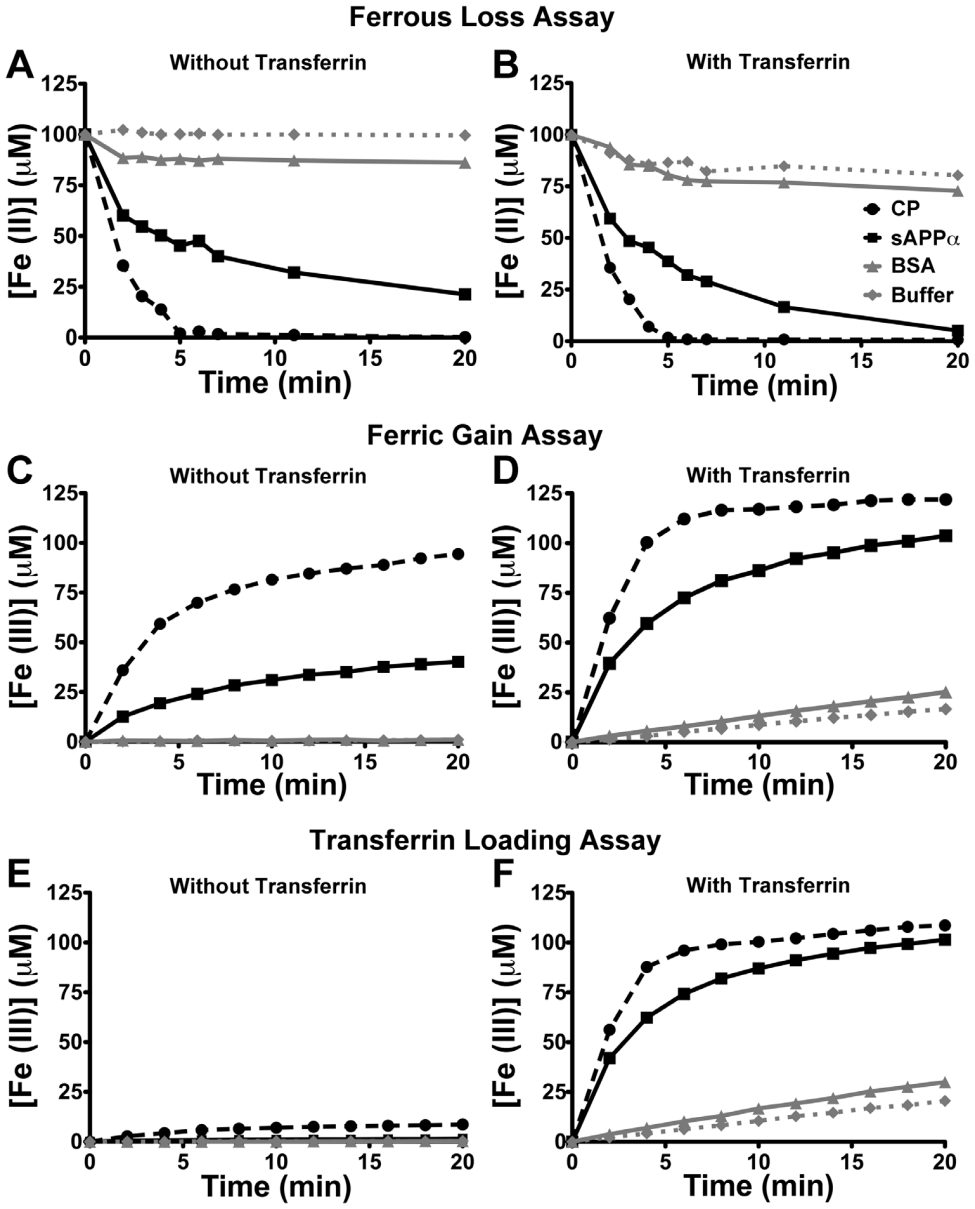
Original preparation of recombinant sAPPα has apparent ferroxidase activity. Using a triplex assay that simultaneously measures kinetically the loss of Fe^2+^ (**A & B**), conversion of Fe^3+^ (**C & D**) and physiologically-relevant loading of Fe^3+^ into transferrin (**E &F**) [22], the apparent oxidase activity of sAPPα (500 nM) (▪) was compared to CP (500 nM) (•), BSA (500 nM) (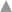) and buffer only (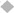) with (**B, D & F**) and without (**A, C & E**) the presence of TF. While activity of the originally prepared recombinant sAPPα was not kinetically comparable to CP in all conditions, activity was greater than BSA used as a control and buffer alone. Assays were run at 26°C in HBS, pH = 7.2+ FAS (100 µM) ± apo-TF (50 µM). Individual data points were mean ± S.E. of 2 experiments, performed in duplicate.

Within a physiologically relevant buffer (HBS, pH 7.2) the abilities of sAPPα and CP to convert Fe^2+^ to Fe^3+^ were measured in the absence and presence of TF. In all conditions the sAPPα (250 nM) preparation was able to decrease Fe^2+^ and increase Fe^3+^ levels compared to control (buffer alone, or with 250 nM BSA) (Fig. 1A–D). Fe^2+^ oxidation proceeded significantly faster in the presence of CP compared to the presence of sAPPα (Fig. 1A–D, Table 1), and the addition of TF to the assay increased the kinetics of both oxidation readouts (Fig. 1B & D, Table 1). Upon measuring Fe^3+^ incorporation by apo-TF, sAPPα and CP were observed to promote comparable generation of holo-TF by the end of the reaction (20 min), similar to that previously reported [2] but kinetically their initial rates of reaction differed (i.e. slope of curve in Fig. 1F, Table 1). Similar results were obtained from the commercially-obtained full-length APP (data not shown).

**Table 1:** Initial rates of reaction for originally purified sAPPα and CP for all 3 outputs in the triplex assay with the absence or presence of TF.

	Without TF		With TF	
	sAPPα	CP	sAPPα	CP
Ferrous loss Fe^2+^(µM)/min	4.90	10.84	7.01	14.24
Ferric gain Fe^3+^(µM)/min	4.84	14.83	14.89	25.09
TF loading Fe^3+^(µM)/min	–	–	20.99	28.11

**Figure 2:**
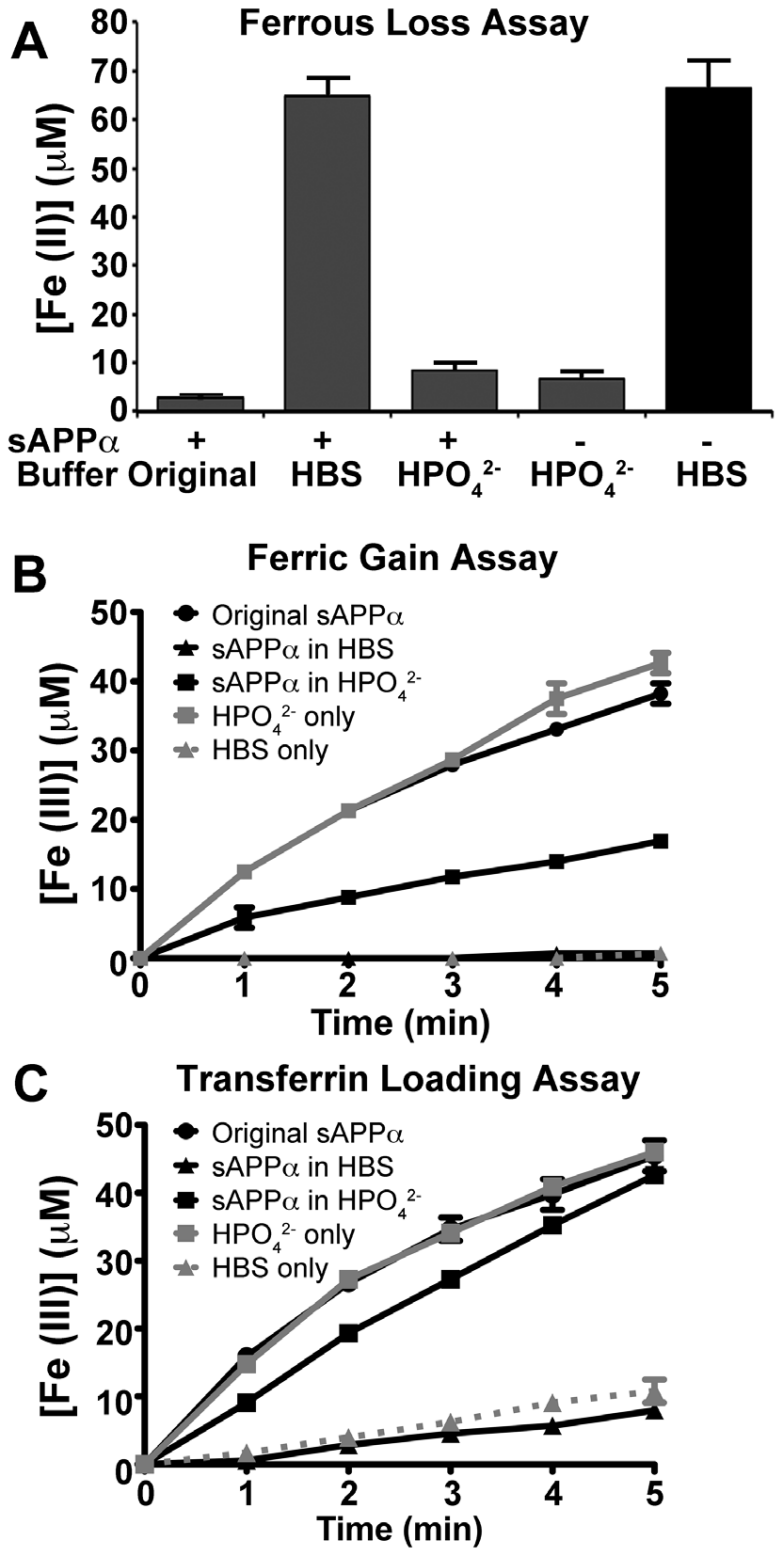
Apparent APP ferroxidase activity derives from the presence of contaminating polyanions. Recombinant sAPPα was originally eluted from phenyl Sepharose with a buffer (50 mM Na_2_HPO_4_, pH = 7.4) that lead to a final assay concentration of HPO_4_^2−^ at 0.5 mM. Buffer exchanging the sAPPα preparation with HBS prior to its use in the assay (▴) eliminated the presence of this trace polyanion and subsequently ablated activity in all measurements of the triplex assay (**A–C**). The original iron oxidation rate of the sAPPα (250 nM) preparation (•) was comparable to Na_2_HPO_4_ alone (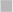), and reintroduction of Na_2_HPO_4_ with sAPPα (▪) by further buffer exchange produced similar activity to that observed with the original sAPPα preparation. Thus, contaminating HPO_4_^2−^ mimicked ferroxidase activity. Individual data points were mean ± S.E. of 2 experiments, performed in duplicate.

We went further to investigate whether a cofactor co-purified with sAPPα was required for the apparent ferroxidase activity. We determined that during the last step of chromatographic purification of sAPPα from yeast, the phenyl Sepharose elution buffer of HPO_4_^2−^ (50 mM) yielded a final concentration in the ferroxidation assay of 0.5 mM HPO_4_^2−^. Prior reports have indicated that polyanions such as phosphate and bicarbonate can promote TF loading [22] [37]–[39]. We had not previously appreciated this 1% contaminant (after dilution with HBS) as significant. We therefore repeated the experiment after removing the residual anions from recombinant sAPPα by size exclusion chromatography with the buffer completely exchanged to HBS, pH 7.2. After this exchange, sAPPα activity was abolished for all outputs of the triplex assay (Fig. 2). Further examination indicated that 0.5 mM HPO_4_^2−^ alone was enough to oxidize Fe^2+^ as well as incorporate Fe^3+^ into TF at a rate comparable to the original sAPPα preparation (Fig. 2). This mimicked ferroxidase activity. Again, similar results were identified with the commercial full length APP (data not shown), which is delivered lyophilized in PBS.

**Figure 3:**
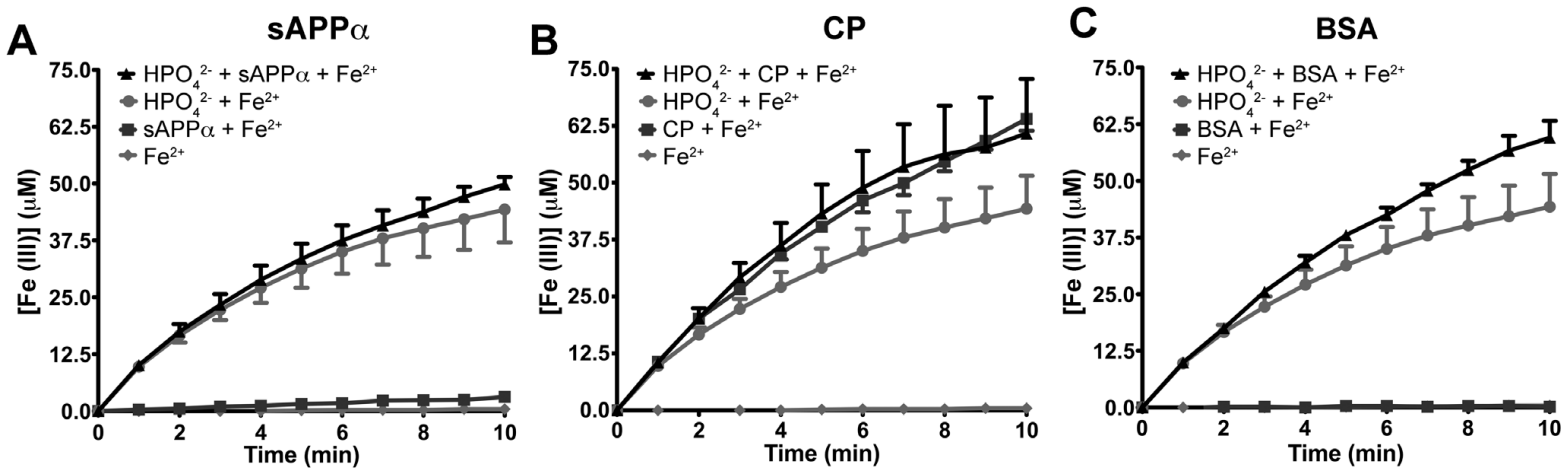
Contribution of phosphate to iron oxidation from preparations of sAPPα, CP or BSA. Within the original triplex assay conditions (HBS, pH = 7.2, 26°C), FeSO_4_ (100 µM) alone had minimal capability to produce Fe^3+^ within 10 min. The presence of Na_2_HPO_4_ (0.5 mM) markedly increased the production of Fe^3+^ over the same period. As previously determined in Fig. 2, sAPPα (250 nM) was unable to facilitate Fe^3+^ production unless in the presence of Na_2_HPO_4_ (**A**), similar to BSA (**C**), whereas CP (250 nM) was unaffected and demonstrated greater formation of Fe^3+^ than Na_2_HPO_4_ alone (**B**). Ferric ion production was measured as previously described [22], [28], [37]. Individual data points were mean ± S.E. of 2 experiments, performed in duplicate.

While the apparent ability of sAPPα and APP to oxidize Fe^2+^ originated from the presence of contaminating HPO_4_^2−^ (Figs. 3A & C), this anion was not required for CP enzymatic activity and had no effect on enhancing the kinetics of the reaction as measured by Fe^3+^ production (Fig. 3B).

### APP increases the stability of Ferroportin on the cell surface

Having excluded ferroxidase activity as being of relevance to the mechanism by which APP facilitates the efflux of intracellular iron [2], [17], we investigated APP interaction with the iron exporter FPN. Our original findings of APP interaction with FPN [2] were recently confirmed using the REXXE-containing peptide fragment of APP, with both this peptide, and exogenous sAPPα, stabilizing surface levels of FPN and promoting iron efflux in human brain microvascular endothelial cells [19].

**Figure 4:**
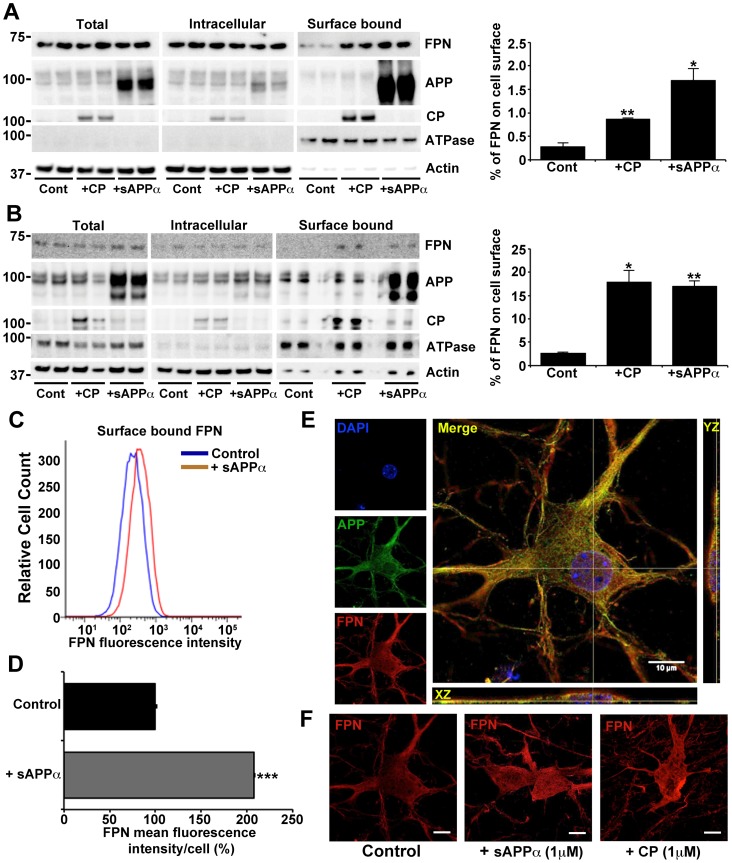
Extracellular sAPPα or CP increases cellular expression of surface ferroportin. FPN location was examined in (**A**) HEK293T and (**B - D**) primary murine neuronal cultures, preincubated with iron (50 µM, 3 h) followed with CP or sAPPα (1 µM, 30 min). Both cell lines have been previously shown to utilize APP to promote iron efflux, and do not express CP [2], [14]. Surface proteins on (**A**) HEK293T cells, and (**B**) primary neurons, were biotinylated to identify changes to endogenous FPN and APP expression on the cell surface, as well as exogenously attached sAPPα or CP. Surface levels of FPN were significantly increased in the presence of CP or sAPPα, despite total levels of FPN remaining unchanged. The graphs show the distribution of FPN when normalized against the β-actin content of the intracellular+surface fractions, and adjusted for protein load. Similar results for FPN distribution were achieved even without adjusting for β-actin (not shown). (**C**) Fluorescence-activated cell sorting of non-permeabilized N2a neuroblastoma cultures preincubated with iron (50 µM, 6 h) confirms an increase in surface expression of FPN, quantified in (**D**), after a 30 min incubation with sAPPα (1 µM) in OptiMEM. (**E**) Deconvoluted confocal microscopy shows overlap of endogenous APP and FPN at the surface of non-permeabilized primary neurons preincubated with iron (50 µM, 3 h), as well as (**F**) increased FPN on the neuronal surface following further treatment with CP or sAPPα (1 µM, 30 min). Endogenous surface FPN was below detection limits in neurons that were not treated with FAS (not shown). Data in (**A**), (**B**) & (**D**) are means ± S.E. of 3 experiments, performed in duplicate. * p<0.05, ** p<0.01 and *** p<0.001 analyzed treatment vs control, by two-tailed t tests. (**C**) is a representative histograms of>10,000 live cells normalized to the control (treated with secondary antibody only) mean signal, set at 10^2^. (**E**) & (**F**) are representative images of a neuron from 2 experiments, performed in duplicate. Scale bar = 10 µm.

Immortalized HEK293T and primary neuronal cultures both utilize APP to promote iron efflux [2], [17]. We tested the effects of a brief exposure to exogenous sAPPα added to the media (1 µM for 30 min) to surface FPN expression in these cells as well as neuroblastoma cells. The cells were pre-incubated with 50 µM iron to achieve detectable starting levels of FPN. Indeed, brief sAPPα treatment increased FPN detected by cell surface biotinylation and FACS (Fig. 4). A similar increase in FPN expression on the cell surface was also observed following incubation with CP (1 µM for 30 min) (Fig. 4A&B), despite this enzyme not being endogenously expressed in either of the cell lines used [2], [14]. The increase of surface FPN induced by CP did not increase cell surface APP. As total FPN expression was unchanged by either sAPPα or CP treatment, the increased cell surface FPN is consistent with stabilization of surface FPN rather than increased FPN production.

Histological assessment of the proximity of APP and FPN on the primary neuronal cell surface was determined by deconvoluted confocal microscopy. Upon the addition of iron, endogenous levels of APP and FPN were colocalized on the surface of non-permeabilized primary neurons (Fig. 4E). Similar to cell surface biotinylation studies, the addition of either sAPPα or CP greatly increased the expression of FPN on the cell surface (Fig. 4F).

**Figure 5:**
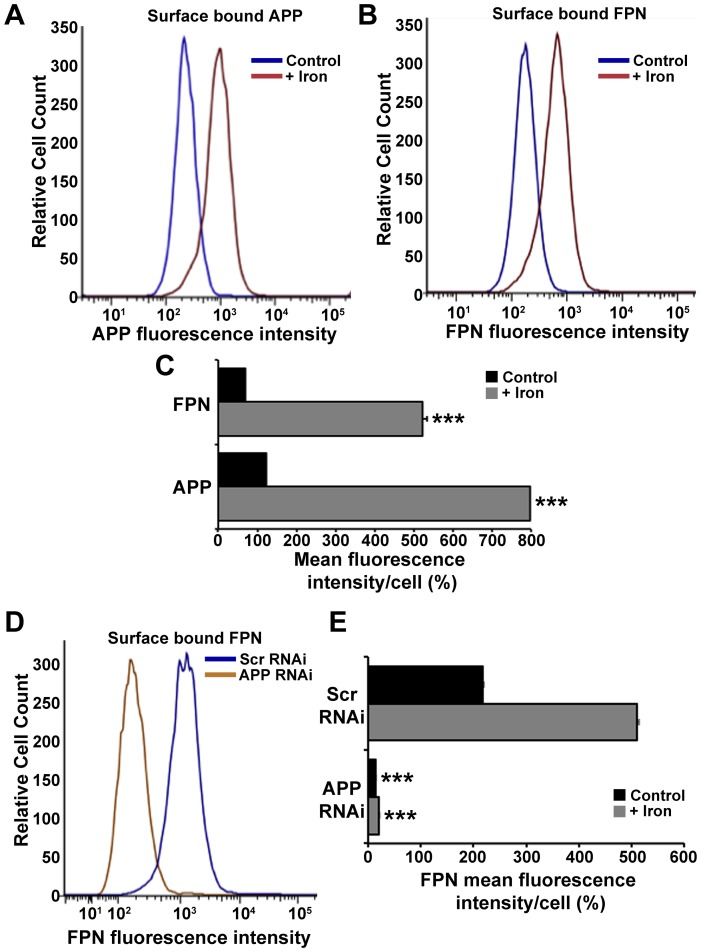
Presence of neuronal surface APP and FPN correlate in response to levels of intracellular iron and the expression of the congruent protein. Fluorescence-activated cell sorting of non-permeabilized N2a neuroblastoma cultures preincubated with iron (50 µM, 6 h) showed an increase in surface expression of (**A**) endogenous APP and (**B**) FPN, quantified in (**C**). (**D**) Despite the previously reported accumulation in intraneuronal iron induced by APP siRNA [2], endogenous surface FPN expression was decreased. (**E**) Surface FPN was exaggerated in the presence of iron (50 µM, 6 h), but not with APP siRNA. (**A**), (**B**) & (**D**) are representative histograms of>10,000 live cells normalized to the control (treated with secondary antibody only) mean signal, set at 10^2^. Quantitation using FlowJo software in (**C**) & (**E**) represent the means ± S.E. of 3 experiments, performed in duplicate. *** p<0.001 in (**C**) analyzed control vs. iron, in (**E**) Scrambled (SCR) vs. APP siRNA, by two-tailed t tests.

The interaction between endogenous APP and FPN was also analysed by FACS of neuroblastoma cells. Treatment with iron (50 µM) induced increased surface levels of FPN, as expected [40], [41], and a concomitant increase in cell surface levels of APP (Fig. 5A–C). In this system, longer duration of iron (50 µM) treatment (for 6 hours rather than just 3 hours treatment for the cell culture data in Figure 4) was optimized to achieve a clear separation of flow cytometry peaks, ± Fe, for FPN, so that RNAi of APP could be tested. When endogenous APP expression was suppressed by siRNA (as confirmed by negligible APP expression after treatment, data not shown), FPN levels on the cell surface were markedly suppressed (Fig. 5D–E) despite the increase in intracellular labile iron that APP suppression can induce [2].

### Culture media alone promotes iron oxidation

We had previously reported that iron efflux from neuroblastoma and primary neurons was promoted by APP and sAPPα in the absence of a ferroxidase enzyme [2]. However, McCarthy *et al* [19] recently reported that while sAPPα promotes iron export, cells require the presence of an exofacial ferroxidase (Fet3). The conditions of these two sets of experiments are noted to differ, as summarized in Table 2; namely, different cells, media, sAPPα concentration and presence of extracellular TF. To reconcile some of these observations we examined the oxidation of Fe^2+^ achieved by the various culture media alone, since if there is sufficient oxidation of Fe^2+^, this could account for iron efflux through Fe^3+^ release from FPN. All media tested contained select proprietary formulations, whose full contents are not published, therefore, there was no way of predicting what amount of oxidation would occur without measuring it empirically.

**Table 2:** Comparison of media conditions relevant for iron oxidation in cell types used to study APP facilitated iron efflux.

Cell Type		hBMVEC [19]	HEK293T [2] & SH-SY-5Y [17]	Primary Neurons [2], [17]
Media		RPMI	OptiMEM	Neurobasal
Inorganic salts	Sodium Phosphate	∼5 mM	∼1 mM	∼1 mM
	Sodium Bicarbonate	∼25 mM	∼25 mM	∼25 mM
	Sodium Chloride	∼100 mM	∼115 mM	∼50 mM
	HEPES	+ (unknown)	+ (unknown)	+ (∼10 mM)
Supplements	Transferrin	−	+ (unknown)	+ (unknown)

**Figure 6:**
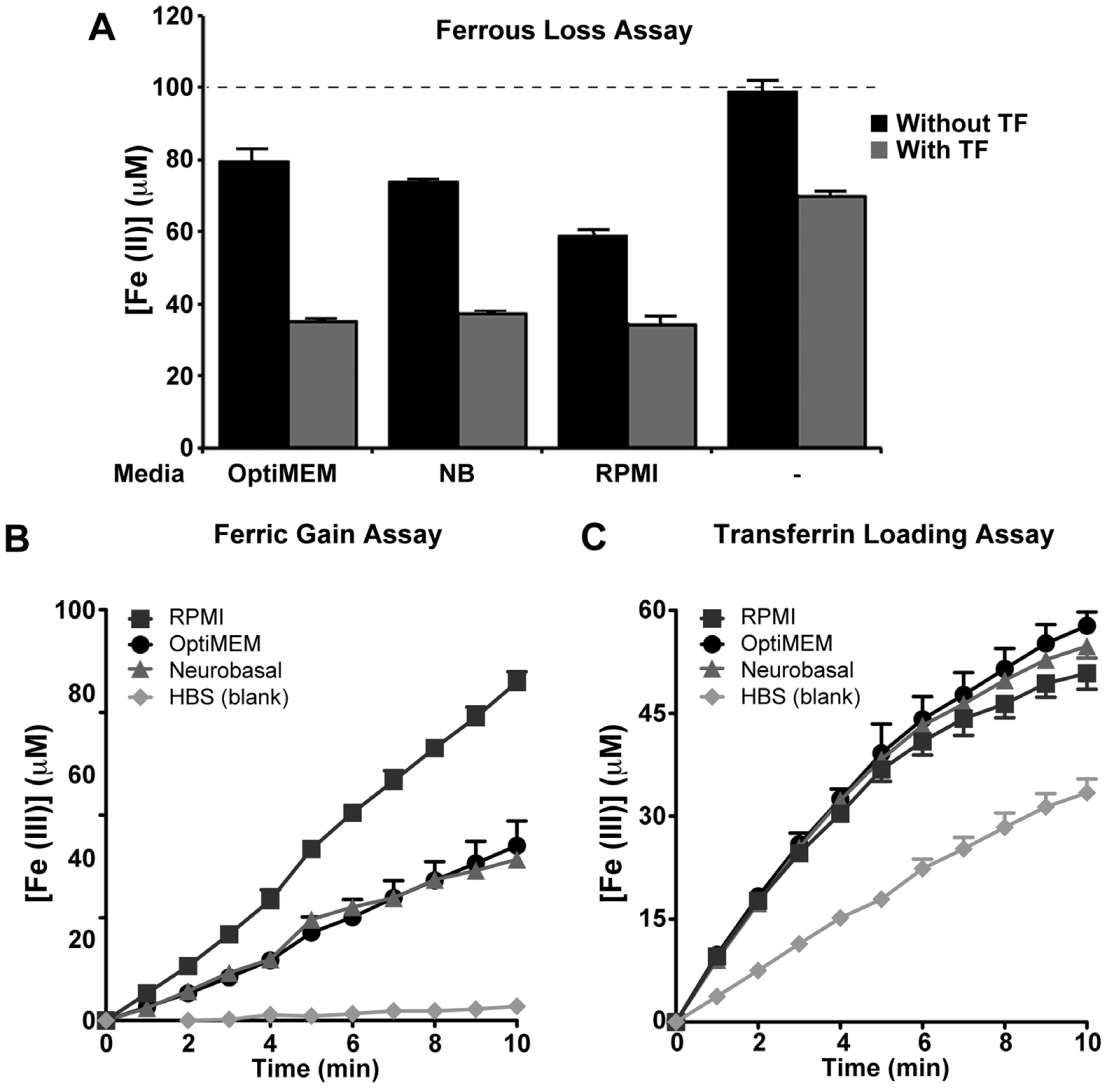
Apparent ferroxidase activity derived from culture medium. Media of interest was tested by the triplex assay to measure loss of Fe^2+^ (**A**), conversion of Fe^3+^ (**B**) and loading of Fe^3+^ into TF (**C**). Without apo-TF, a one in five dilution of OptiMEM, neurobasal (NB) and RPMI-1640 (RPMI) media oxidized Fe^2+^ to Fe^3+^ (**A & B**). The addition of apo-TF (50 µM) promoted Fe^2+^ oxidation by all media (**A**), and had comparable ability to load Fe^3+^ onto TF (**C**). Assays were run at 26°C in HBS, pH = 7.2+ FAS (100 µM) ± apo-TF (50 µM). Individual data points were mean ± S.E. of 2 experiments, performed in duplicate.

Indeed, all three commercial media utilized by the two studies were able to induce markedly greater Fe^2+^ oxidation than HBS pH 7.2 (Fig. 6A&B). While this could account for our observation that APP and sAPPα promotes iron efflux without the presence of a ferroxidase, McCarthy *et al* [19] reported that the ferroxidase (Fet3) promoted efflux above the levels achieved by sAPPα alone. McCarthy *et al* [19] did not supplement with extracellular TF in their media, whereas we did (Table 2), so we examined the impact of TF on the oxidation induced by each media. Indeed, the presence of TF augmented oxidation by each media (Fig. 6A), and the loading of Fe^3+^ into TF was comparable in all media (Fig. 6C), demonstrating that TF dominates the reaction, and can limit the amount of oxidation that is achieved by the polyanionic environment. Therefore, the presence of TF in the cell culture media is predicted to be a dominating influence on the efflux of iron, and may be the reason why McCarthy *et al* [19] only saw sAPPα promoting iron efflux in the presence of a ferroxidase (but the absence of TF), whereas we [2] observe sAPPα and APP promoting iron efflux in the absence of a ferroxidase (but the presence of TF).

## Discussion

Using a recently reported adaptation of existing assays for measuring Fe^2+^ oxidation in physiologically relevant buffers [22], we found that our previously-reported oxidase activity of APP actually originated from contaminant phosphate that co-eluted during protein purification. Despite this, our data confirm the previous findings [2], [17], [19] that APP decreases intraneuronal iron by stabilizing FPN on the neuronal cell surface. McCarthy *et al* [19] recently reported that the presence of an extracellular ferroxidase (Fet3) was necessary for sAPPα-assisted iron export in endothelial cells. However, our previous data indicate that the facilitatory role of APP in effluxing neuronal iron did not require ferroxidase activity or an endogenous multicopper ferroxidase, as neither were detectably present in the original neuronal culture studies [2]. However, similar to previous iron efflux studies [12] both TF and polyanions such as phosphates and bicarbonates were present in the media used (OptiMEM and Neurobasal), and therefore may have promoted Fe^3+^ loading into TF [22], [42] as well as the chemical oxidation of Fe^2+^ released from the exofacial surface of FPN. It remains to be determined whether an exogenous ferroxidase will augment the neuronal iron efflux that is promoted by sAPPα in the same manner as shown with endothelial cells. Nonetheless, rapid oxidation of Fe^2+^ is evident in the presence of apo-TF and phosphate [43] and we have determined that APP and sAPPα can promote Fe^3+^ efflux in a suitable polyanionic/TF environment without the presence of an extracellular ferroxidase [2]. This use of a non-transferrin bound iron efflux route with RPMI media in the absence of TF (as in [19]), may explain the variable outcomes obtained between studies.

Here we have attempted to appraise the involvement of neuronal APP in the 3 known criteria of iron export; namely, stabilization of ferroportin on the cell surface (by APP), iron oxidation (by the media), and loading of TF (by polyanions). Taken together, we hypothesize that under conditions requiring rapid iron efflux, or within an environment where Fe^2+^ oxidation by polyanions is limited, a ferroxidase enzyme may be needed to assist iron efflux. Such a circumstance would be where pH and the anion gap are altered, such as within a hypoxic environment and hypophosphatemia, or when apo-TF and/or APP/sAPPα are in limited supply, such as with anemia of chronic inflammation and Alzheimer’s disease respectively [44]–[47]. Despite CP not being endogenously expressed in neurons, its recruitment from other cell types may still promote FPN persistence on the neuronal cell surface (Fig. 4) and release of Fe^3+^ from FPN to facilitate iron efflux [19]. Our previous evidence illustrating an increased interaction between FPN and CP in brains of APP knockout mice [2] supports this hypothesis.

We conclude that despite no current evidence of endogenous ferroxidase expression in a neuron, APP in a physiological environment still facilitates the efflux of iron in the presence of TF. We hypothesize that by APP stabilizing FPN on the cell surface, the anionic interstitial environment oxidizes Fe^2+^ whereupon Fe^3+^ is incorporated into TF. We propose that this process for iron efflux is predominant where a ferroxidase is not present, but can be superseded in conditions of stress where a ferroxidase is used to increase the efflux of iron with minimal ROS production. It is important to consider that, while neurons have high metabolic activity and therefore generate more ROS, the iron elevation in brain only causes neurodegeneration with age [48], [49]. This phenotype is accelerated when either APP or CP is genetically ablated in mice [2], [50] and may be a contributory pathogenic mechanism in human diseases associated with each protein, such as Alzheimer’s disease [51], [52], Parkinson’s disease [53] or aceruloplasminemia [54], [55].
